# A Knowledge and Practice Survey Among Dentists in Saudi Arabia Analysing Myths and Misconceptions in Dentistry and Oral Surgery: What Do Dentists Believe?

**DOI:** 10.7759/cureus.36625

**Published:** 2023-03-24

**Authors:** Maisa Al-Sebaei, Mohammed A Sindi

**Affiliations:** 1 Oral and Maxillofacial Surgery, King Abdulaziz University (KAU), Jeddah, SAU; 2 General Dentistry, Ministry of Health, Jeddah, SAU

**Keywords:** dentistry and anticoagulants, local anesthesia, odontogenic infections, myths and misconceptions, dentistry and pregnancy, dental radiology, oral surgery, dental curriculum, dental education, survey study

## Abstract

Introduction

To ensure safe and effective practice, dental practitioners must stay up-to-date with all scientific updates involving their profession. In this regard, many outdated myths and misconceptions may be persistently believed and practiced. This study aimed to examine dental misconceptions circulating among dentists in Saudi Arabia.

Methods

An electronic survey was administered to Saudi Arabian dental practitioners classified and registered with the Saudi Commission of Health Specialties. It collected their demographics, career and experience details, and responses to 16 questions that targeted different myths. Logistic regression was used to analyze factors associated with their knowledge.

Results

A total of 519 dentists answered the survey, of which 54% were male with a mean age of 32 ± 9 years and a mean practice of 7 ± 8 years. More than half (57%) practiced general dentistry. In most (69%) of the questions, 40% of the respondents answered incorrectly. The proportion of incorrect answers to some questions reached 62%. Years of teaching, years in practice, and doctor rank had no association with the knowledge score. Conversely, the type of practice and specialty had multiple statistically significant associations (p < 0.05).

Conclusion

This study shows that many myths, despite being debunked for more than 20 years, are still circulating among Saudi Arabian dentists, including many young dentists. Academic institutions must urgently address these concepts and the science that disproves them; dentists must implement up-to-date, evidence-based knowledge in their practice.

## Introduction

Studies assessing dental professionals' knowledge and practices help determine future learning priorities for dentists and contribute to the systematic development of the profession. In the recent past, most studies assessing the knowledge and practices of dental professionals have predominantly focused on advancements in single subjects, such as biomedical waste management, infection control, antibiotics stewardship, oral cancers, special populations, and adverse drug reactions [1-5]. In contrast, only a few studies have comprehensively and simultaneously evaluated the knowledge and practices of dentists in more than one of the above subject areas and other practice areas that have witnessed several evidence-based and potentially practice-changing updates and evidence. In dentistry and oral surgery, many concepts have changed and evolved over the years based on the advancements in evidence-based dentistry. These concepts changed how dentists perform certain techniques or manage disease processes. The general or specialized dentist must be informed of the emerging evidence and apply it to daily practice. Therefore, our study aimed at evaluating certain concepts that have changed in the past 10-20 years to assess whether community-based practicing Saudi Arabian dentists still adhere to outdated management techniques and whether some myths and misconceptions still exist among them. This study also aimed to establish an association of the dentists' knowledge with the number of years in practice, rank, and type of practice. This article will present the survey results, followed by a conceptual discussion based on the latest literature.

## Materials and methods

This cross-sectional study was approved by the Research Ethics Committee at King Abdulaziz University Faculty of Dentistry (REC-KAUFD), Jeddah, Saudi Arabia. The target subjects for this study were general and specialized dentists working in major cities in Saudi Arabia in private offices, government sectors, and academic institutions. Inclusion criteria were dentists working in the government or private sectors with at least a Bachelor of Dental Surgery degree or equivalent with one or more years of experience after graduation and registered and classified with the Saudi Commission for Health Specialties. Exclusion criteria included dental interns, students, and assistants.

Survey

The survey was prepared and revised by Dr. Maisa Al-Sebaei, an associate professor practicing oral and maxillofacial surgery (OMFS) and academia for over 25 years. The survey was then validated by content and face validation, with the help of other oral and maxillofacial consultants who reviewed the questions and the scientific basis behind all domains. Lastly, the survey underwent criterion validation (predictive outcome) with the help of a consultant in dental public health and biostatistics. All of them advised no changes to the questions or the survey structure. The survey responses were obtained over six months, from March 2021 to November 2021. It was sent electronically using the database of dentists registered at the Saudi Commission for Health Specialties. The survey cover explained the aim of the study, collected some relevant demographic information, and included consent for voluntary participation. Dentists participated in the survey voluntarily and were assured of their anonymity and the confidentiality of their responses. No personal data were collected. The survey sought information on the following items: demographic data: gender, age, years of experience, specialty, type of practice (government, private, or academic), current rank in the Saudi Commission for Health Specialties, the region in which undergraduate and postgraduate education was obtained, and years of teaching experience, if applicable. Special attention was paid to the recording of the specialty due to the effect that certain specialties could have on the outcome of different domains. The body of the survey consisted of 16 questions in seven domains (odontogenic infection, antibiotic therapy, anticoagulants, local anesthesia, pregnancy, infection control, and tooth crowding), for which the participant was instructed to answer "True or False." All the questions were common misconception statements; therefore, the correct answer was always "False" (Table 1). For each respondent, a novel knowledge score was calculated for each domain based on the correct answer to each question and tested for an association with the following variables (type of practice, specialty, rank and years in practice, and years of teaching experience).

**Table 1:** The survey questions consisted of 16 questions in seven different domains for which the participants were instructed to answer "True or False."

Domain	Question (All correct answers are false)
Odontogenic infection	You must NOT extract lower third molars in the presence of pericoronitis.
	An extraction CANNOT be performed without complications in the presence of an odontogenic infection.
	An incision and drainage should NOT be performed in the cellulitis stage of an odontogenic infection. (It is required to wait for the cellulitis to become a pus-localized abscess to perform an incision and drainage).
	Performing an extraction or root canal during the cellulitis phase of an odontogenic infection spreads the infection.
Antibiotic therapy	You must prescribe a post-operative antibiotic to all patients who undergo third molar removal procedures.
	All diabetic patients must be administered pre-operative antibiotics before extraction.
	Pre-operative infective endocarditis antibiotic prophylaxis must be administered to all cardiac patients.
Anticoagulants	Aspirin must be discontinued before any tooth extraction.
	Continuous anticoagulant therapy must be discontinued before any dental surgery.
Local Anesthesia	Local anesthesia with epinephrine must NOT be administered to patients with coronary artery disease and hypertension.
	When giving an inferior alveolar nerve block, a negative aspiration ensures a non-vascular injection.
	It is dangerous to give bilateral inferior nerve blocks because the patient will "swallow his tongue"(i.e., cause airway obstruction).
Pregnancy	It is NOT safe to subject pregnant patients to intraoral radiographs.
	It is NOT safe to administer local anesthesia with 1:100,000 epinephrine to a pregnant patient.
Infection Control	For proper infection control, patients with known hepatitis and HIV should be treated as the last patient of the day.
Tooth Crowding	Wisdom teeth eruption causes teeth crowding.

Data analysis

Data from the questionnaires were entered into a computer database and analyzed using R programming language version 3.6.3. Categorical data are presented as frequencies, using percentages. Continuous data are presented as mean and SD. Logistic regression was performed to analyze the factors (rank, type of practice, specialty, years in practice, and years of teaching experience) associated with the knowledge score. The consultant rank, academic practice, and OMFS were used as references for the comparisons. A p-value < 0.05 was considered significant.

## Results

A total of 519 dentists responded to the survey. The age range of the respondents was 25-70 years, with a mean age of 32 ± 9. The mean years in practice were 7 ± 8 years, with the lowest at one year and the highest at 45 years. The mean number of years in teaching was 2.5 ± 5.6, ranging from 0 to 31 years. The demographics of the sample are presented in Table 2.

**Table 2:** Demographics of the study sample. OMFS: Oral and maxillofacial surgery.

	N (%) out of 519
Gender	
Females	237 (46%)
Males	282 (54%)
Rank	
General Dentist	311 (60%)
Consultant	106 (20%)
Specialist	102 (20%)
Specialty	
General Dentistry	294 (57%)
Restorative/Prosthodontics	64 (12%)
OMFS	54 (10%)
Endodontics	35 (6.7%)
Periodontics	29 (5.6%)
Pediatric Dentistry	22 (4.2%)
Orthodontics	13 (2.5%)
Oral Path/Oral Medicine	8 (1.5%)
Type of Practice	
Government	312 (60%)
Private	124 (24%)
Academic	83 (16%)
Undergraduate Degree	
MENA Region	516 (99%)
UK/Europe	3 (0.6%)
Postgraduate Degree	
None	290 (56%)
MENA Region	137 (26%)
North America	59 (11%)
UK/Europe	33 (6.4%)
Region	
Makkah	364 (70%)
Riyadh	131 (25%)
Others	24 (5%)

**Figure 1:**
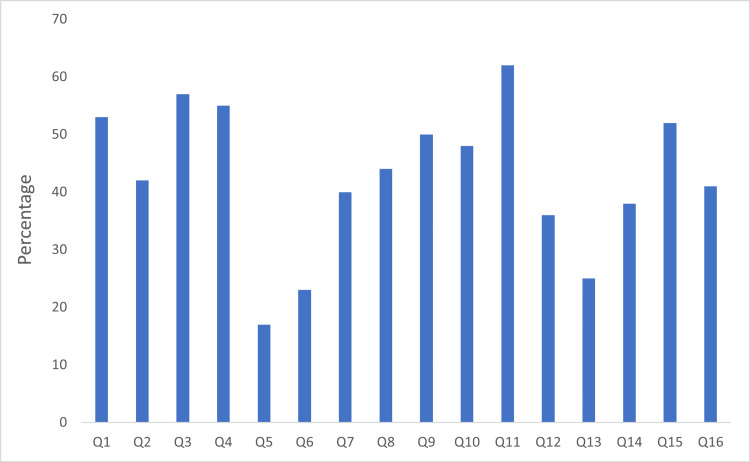
The percentage of dentists who responded “True” to the stated misconceptions. Q1-4: Odontogenic Infection, Q5-7: Antibiotic Therapy, Q8-9: Anticoagulant Therapy, Q10-12: Local Anesthesia, Q13-14: Pregnancy, Q15: Infection Control, Q16: Teeth Crowding.

The dentists' response to each question is shown in Figure 1. The graph shows the percentage of dentists who did not answer the questions correctly and believed the stated misconceptions were true. In 11 out of 16 questions, 40% of the respondents answered incorrectly. The questions with the highest percentage of incorrect answers (62%) were Q11 (negative aspiration), followed by Q1, Q3, and Q4 (>50%), which fall into one domain (odontogenic infection). On the other hand, the questions with the lowest percentage of incorrect answers among the dentists (<25%) were Q5 and Q6 (antibiotic therapy) and Q13 (pregnancy and radiographs).

Across all the domains, the rank, years in practice, and years of teaching were not associated with the knowledge score. Compared to academics, dentists in the private sector had a lower knowledge score in five out of seven domains (Q1 to 14); odontogenic infection, antibiotic therapy, anticoagulant therapy, local anesthesia, and pregnancy. Regarding specialty, the orthodontics and oral pathology/oral medicine specialized dentists had consistently similar knowledge as OMFS across all the domains. Contrarily, endodontics, general dentistry, and restorative/prosthodontics specialties had lower knowledge than OMFS in three or more domains. Table 3 presents the p-values of the logistic regression model used on the factors and the knowledge scores.

**Table 3:** P-values for the logistic regression model used to identify the factors associated with the knowledge score. * denotes statistically significant at *p* <0.05; ** is the reference used for comparing the factors. OMFS: Oral and maxillofacial surgery.

Domain	Odontogenic Infection	Antibiotic Therapy	Anti-coagulation	Local Anesthesia	Pregnancy	Infection Control	Tooth Crowding
Questions	Q1-4	Q5-7	Q8-9	Q10-12	Q13-14	Q15	Q16
Rank							
Consultant**							
Specialist	0.317	0.331	0.995	0.277	0.837	0.166	0.628
GP	0.633	0.253	0.117	0.338	0.439	0.656	0.806
Type of Practice							
Academic**							
Government	0.107	0.164	0.390	0.082	0.327	0.358	0.659
Private	0.000*	0.000*	0.000*	0.000*	0.001*	0.937	0.459
Specialty							
OMFS**							
Endodontics	0.001*	0.312	0.008*	0.028*	0.464	0.861	0.581
Orthodontics	0.130	0.650	0.095	0.115	0.904	0.832	0.813
Periodontics	0.004*	0.198	0.001*	0.612	0.866	0.154	0.683
General Dentistry	0.000*	0.047	0.066	0.004*	0.039*	0.855	0.162
Pediatric Dentistry	0.009*	0.204	0.009*	0.214	0.162	0.932	0.237
Oral Path/Oral Medicine	0.134	0.468	0.060	0.566	0.842	0.453	0.748
Restorative/Prosthodontics	0.000*	0.024*	0.000*	0.016*	0.045*	0.562	0.399
Years in Practice							
	0.782	0.469	0.367	0.216	0.943	0.065	0.892
Years of Teaching							
	0.572	0.401	0.882	0.118	0.413	0.407	0.651

## Discussion

The authors focused on determining the prevalence of misconceptions regarding various dental practices in the community, irrespective of educational level, years of experience, or specialty. Our results indicate that rank and years of experience were not statistically significant variables. However, dentists in private practice had a lower knowledge score than those in academia. This can be attributed to the nature of the academic practice, where practitioners are updated with the latest literature due to their involvement in teaching and clinical supervision. The significance of dental specialty as a factor in knowledge scores varied among specialties. For example, endodontics showed similar knowledge scores to OMFS in domains such as antibiotic therapy, pregnancy, infection control, and tooth crowding. At the same time, restorative dentists and prosthodontists had similar knowledge scores to OMFS in infection control and tooth crowding. Only two specialties, orthodontics and oral medicine/oral pathology showed similar knowledge scores to OMFS in all domains. There is considerable overlap between oral medicine/oral pathology and OMFS, as both work in hospital settings compared to other dental specialties that primarily practice in dental clinics. Additionally, theoretical and practical training in oral medicine/oral pathology and OMFS can focus on different subjects compared to other dental specialties, such as general human diseases and conditions. Interestingly, orthodontists scored similarly to OMFS, which may be because orthodontics is a unique specialty that rarely deals with clinical scenarios like antibiotic therapy and infection control. This suggests that participating orthodontists' knowledge is at a basic undergraduate level that has not been modified by clinical practice. In general, differences in dental specialty knowledge scores may be due to diversity in training, educational background, nature of the practice, and length. However, these misconceptions still exist regardless of dental specialty. Correcting misconceptions and misinformation can be quite challenging. In the following section, each domain examined in our study will be reviewed, and evidence will be provided for the correct information about the concept.

Odontogenic infection

Odontogenic infections arise from dental caries, decay, and periodontal disease. These infections tend to have local destructive effects and could, in some instances, develop to affect distant parts of the body (i.e., Ludwig's angina, infective endocarditis (IE), and necrotizing fasciitis) [6]. In this study, nearly half of the participants did not have the correct concept of odontogenic infection management. There is no evidence that the extraction of a tooth or performing a root canal in the face of an odontogenic infection will cause spread [7]. Scientific papers published as far back as 1968 [8] and 1975 [9] provided evidence that extracting teeth in the presence of infection not only did not spread the infection but also contributed to a rapid resolution compared to those whose extractions were performed after the resolution of the infection. Similarly, the extraction of lower third molars in the presence of pericoronitis goes beyond being not contraindicated to being associated with faster and better recovery and shorter hospital stay [10]. The only reason some dentists or surgeons may wait to extract in the presence of pericoronitis is the inability to obtain proper local anesthesia in the face of the infection or the difficulty of performing the extraction due to trismus [11]. The treatment of the cause behind the odontogenic infection must be a priority for all dentists, regardless of the stage of the infection. There is no evidence that removing the cause by extraction or performing an incision and drainage spreads the infection [10]. On the contrary, whether the proposed treatment is an extraction, root canal treatment, or incision and drainage, the evacuation of any trapped pus and the introduction of oxygen to infections that consist primarily of anaerobic bacteria, mainly Streptococcus viridians and Staphylococcus aureus, will provide excellent support to the host's defense in fighting off the infection and the possibility of reaching complete resolution faster [12-13].

Antibiotics

Our study revealed that antibiotic practice in terms of pre-operative prescriptions for diabetic patients and routine post-operative antibiotics for extraction does not seem to be a major area of misconception, as more than 75% of the dentists answered correctly. However, the more concerning finding in this domain was that 40% of dentists need to understand the indications of IE prophylaxis. Medical prescriptions in dentistry are usually limited to antibiotics and analgesics. The use of antibiotics in dentistry is highly regulated. The American Dental Association (ADA) publishes periodic guidelines for using antibiotics in dentistry, pre-operatively and post-operatively [14]. Excessive and unnecessary prescription of antibiotics may lead to burdening consequences, such as methicillin-resistant Staphylococcus aureus (MRSA) and multi-drug resistant (MDR) pathogens, which pose a serious and significant threat to human health [15]. In 2016, the ADA and American Academy of Orthopedic Surgeons said that prophylactic antibiotics are not recommended before dental procedures to prevent prosthetic joint infections [16]. In 2021, the American Heart Association issued a statement [17] confirming that there are no changes to their original IE prophylaxis in dental procedures guidelines that were originally published in 2007, which state that prophylactic antibiotics for IE should only be given to patients with prosthetic cardiac valves, prosthetic materials used to repair cardiac valves, positive history of IE, cardiac transplant patients with regurgitations caused by structurally abnormal valves, unrepaired cyanotic congenital heart disease, and any repaired congenital heart defects with residual shunts or valvular regurgitations [18]. The ADA does not recommend any prophylaxis for diabetic patients. Lastly, a Cochrane review by Lodi G et al., published in 2012, concluded that while antibiotics may contribute to reduced risk of infection, the side effects are usually more pronounced. There was no evidence that post-operative antibiotics prevented swelling, fever, and mouth-opening restrictions in patients who underwent third-molar extraction procedures [19].

Anticoagulation

In this study, nearly half of the dentists responded that aspirin and anticoagulation therapy should be discontinued before tooth extraction. This is concerning because the body of evidence to support continuing both is huge [20-24]. Anticoagulants are a group of medications prescribed to patients with a history of stroke, myocardial infarction, embolism, coronary artery procedures, and others. They help prevent blood clotting to various degrees and prevent potential recurrence or complications of blood coagulation conditions. This may interfere with wound healing in some cases. Anticoagulants can work on the platelet surface to prevent platelet adhesion, such as the case with Aspirin (acetylsalicylic acid) and Plavix (clopidogrel bisulfate). They can work on factors of the coagulation cascade, such as vitamin K antagonists (i.e., warfarin), thrombin inhibitors (i.e., heparin), and others [20]. Regarding patients on Aspirin, the consensus and recommendation are to continue Aspirin therapy if the patient is undergoing simple tooth extraction; this is backed by a very low incidence rate of bleeding complications that, even when they occur, can be controlled efficiently by utilizing local hemostatic measures [21]. However, when the patient is on warfarin, it is crucial to verify that the patient's international normalized ratio (INR) is within the acceptable range (≤4, and preferably ≤3), and in case the INR is above these values, a consultation with the primary treating physician is warranted [22-24]. The risk of discontinuing the anti-platelet or anticoagulation and having a thrombotic event is much higher than bleeding from a tooth socket. There were no reported cases of serious bleeding from tooth extraction that resulted in transfusion or severe outcomes. Many new types and classes of anticoagulation medications have been recently approved and introduced to the consumer market, such as novel oral anticoagulants (NOACs) and target-specific oral anticoagulants (TSOACs). Both classes are relatively new, and their drugs may act in multiple ways or target different steps or factors in the coagulation cascade. Multiple studies have suggested protocols and guidelines to treat patients on NAOCs and TSOACs who may need dental surgeries and procedures [25-28]. Despite that, there is still mist surrounding the newly introduced and updated medications, which can pose a hurdle in providing necessary treatment. A competent dentist should always prioritize the safety of his patients, whether by searching for updated treatment protocols specific to the patient's drugs and conditions or by consulting the patient's treating physician.

Local anesthesia

According to our findings, nearly two-thirds of dentists need a clear idea of proper aspiration techniques. In contrast, one-third must be appraised that bilateral inferior alveolar nerve blocks (IANBs) do not cause airway obstruction. An upper airway obstruction caused by the tongue is considered mechanical, such as when the tongue obstructs the airway by falling posteriorly secondary to the head position in an unconscious or semi-conscious patient [29]. Thus, opening the airway by repositioning the jaw and moving the tongue forward is an AHA guideline for Basic Life Support [30]. An IANB is an injection that targets the inferior alveolar nerve as it enters its canal. It can block the sensation in all teeth in the lower ipsilateral quadrant. A bilateral IANB is sufficient to block the sensation in all lower teeth, including a few surrounding soft tissue structures [31-33]. There is minimal evidence of the risks associated with bilateral IANB; the nerves that may collaterally be subjected to anesthesia during IANB due to their anatomic location are the lingual nerve, which contains no motor fibers, and the hypoglossal nerve, which supplies to the hypoglossal muscle. At the same time, the primary motor function of the tongue is provided by the hypoglossal nerve (IX), which is not anesthetized during proper IANB. As such, the risk of completely numbing the motor function of the tongue, causing the patient to "swallow his tongue," is not scientifically plausible [32]. Local anesthetics come in two variations, plain and with a vasoconstrictor. The vasoconstrictor helps prolong the anesthesia's effect while decreasing potential bleeding in the area. However, epinephrine may increase heart rate and blood pressure due to its effect on alpha receptors [34]; therefore, patients with cardiovascular conditions may risk developing adverse reactions with high doses of epinephrine. However, it has been proven that 0.04 mg of epinephrine has a negligible effect on heart rate and blood pressure [35]. Therefore, a maximum dose of 0.04 mg of vasoconstrictor is recommended for patients with cardiac risk [36]. If the dentist requires additional doses, this limit can be overcome by using plain cartridges or by ensuring that the half-life of epinephrine has passed. Another common misconception in local anesthesia is that negative aspiration ensures a non-intravascular injection. Adverse reactions can also appear in non-cardiac patients in cases of intravascular injections. This is caused when the tip of the needle is inside a vessel. Although it is a common practice to perform negative aspiration before the administration of the local anesthesia during IANB, this does not guarantee proper placement of the tip of the needle for multiple reasons, such as anatomical variations, incorrect technique, movement after the aspiration, aspiration of the vessel wall, and tissue resistance against the bevel of the needle. Sound anatomical knowledge and proper employment of the injection technique or alternative techniques, such as rotating the needle 180 degrees to aspirate in two planes, can contribute to a lower failure rate [31, 37].

Pregnancy

Our results indicate that most dentists have the correct concepts when administering local anesthesia or taking radiographs in pregnant patients. When prescribing or administering drugs to a pregnant woman, it is essential to have profound knowledge of the drugs' pharmacokinetics. If the medication can cross the placental barrier, then it may pose risks to the developing fetus. The FDA's pregnancy risk classification and categories are a handy reference to drug safety. Moreover, a fetus can be susceptible to radiation, especially during the first and third trimesters [38-40]. The FDA's pregnancy risk classification and categories consist of five classes (A, B, C, D, and X), with drugs in category A having unlikely possibilities of harming the fetus after controlled studies in pregnant women and drugs in category X having confirmed fetal abnormalities and potential risks that even extend to the mother sometimes. Lidocaine is considered the safest local anesthetic that can be used in pregnant women, being in category B. Mepivacaine and bupivacaine are both in category C, meaning that they should only be used when benefits outweigh the risks of harmful adverse reactions and effects [33]. Despite the necessity of asking any suspected woman about her pregnancy, obtaining intraoral radiographs for pregnant women is considered safe when their condition mandates it. Still, all precautions should be exercised, including minimizing the radiation by using high-speed films, using lead aprons, and obtaining the radiograph in the second trimester instead of the first or the third, if that was an option [40].

Infection control

It was apparent from the dentists' responses that approximately half of them believe that known hepatitis and HIV patients must be treated at the end of the day. However, no special precautions other than universal precautions are mandated in the dental office for known hepatitis or HIV patients. Standard universal precautions for infection control must be followed for all patients visiting the dental office as per ADA and CDC guidelines; these include protective eyewear, proper attire, mask, proper handling of sharps, etc. [41-42]. No evidence in the literature suggested that hepatitis or HIV, both of which are transmitted through blood-to-blood transmission, require an extra level of disinfection or decontamination or to be treated as the last patient of the day, especially considering the lack of evidence of their transmission through saliva [43-44].

Tooth crowding

Our findings show that fewer than half of the dentists believe that the eruption of wisdom teeth will cause tooth crowding. Many dentists and dental professionals worldwide believe that the eruption of the lower third molars can lead to anterior crowding in the mandibular teeth due to space restriction and the "pushing effect" of the third molars [45]. Despite this widespread controversy, randomized clinical trials and high-level, low-bias systematic reviews have demonstrated that the principal concept that the eruption of lower third molars can cause anterior crowding in the mandibular teeth is unjustified and cannot be established. No solid or consistent evidence supports this theory [45-46].

Limitations

This survey had some limitations. This was a cross-sectional survey and is prone to various types of bias associated with observational studies. Additionally, this study devised a novel knowledge score for comparisons between dentists. This knowledge score used in this study needs to be validated in future studies. In addition, more research is required to replicate our findings. Furthermore, the reasons for the persistence of misinformation despite scientific debunking and the remedial strategies could not be examined in this electronically conducted survey, which could be the subject of future research.

## Conclusions

In conclusion, this survey aimed to assess the magnitude of some common misconceptions in dentistry and oral surgery. Our results suggest that general practice dentists and specialized dentists in Saudi Arabia still believe in and are practicing some outdated concepts that require debunking and that, regardless of specialty, these misconceptions are still widespread. Furthermore, a priority intervention is warranted for dentists working in private practice as they had a lower knowledge score than those working in academic or government institutions. Additionally, dentists must implement up-to-date, evidence-based knowledge in their practice and seek evidence-based information throughout their careers through their respective specialty associations and by attending continuing education lectures and workshops. Academic institutions must urgently address these concepts and the science that disproves them. Lastly, more research is needed to assess the extent of misinformation among dental practitioners and determine the optimal remedial strategies.
